# Time and cost burden associated with docetaxel in patients with metastatic castration-sensitive prostate cancer initiating an androgen receptor pathway inhibitor-based regimen

**DOI:** 10.3389/fonc.2025.1650378

**Published:** 2025-09-26

**Authors:** Daniel Sentana-Lledo, Arjun Gupta, Carmine Rossi, Sabree Burbage, Jill Korsiak, Lilian Diaz, Gordon Wong, Dominic Pilon, Ibrahim Khilfeh, Alicia K. Morgans

**Affiliations:** ^1^ Dana-Farber Cancer Institute, Boston, MA, United States; ^2^ University of Minnesota Medical School, Minneapolis, MN, United States; ^3^ Analysis Group, Inc., Montréal, QC, Canada; ^4^ Johnson & Johnson, Horsham, PA, United States

**Keywords:** androgen receptor antagonists, chemotherapy, hormone receptor agonists, mCSPC, prostate neoplasms, time burden, triplet therapy

## Abstract

**Background:**

The triplet combination of chemotherapy (docetaxel), androgen receptor pathway inhibitors (ARPI), and androgen deprivation therapy has recently become a recommended approach to treat metastatic castration-sensitive prostate cancer (mCSPC). This study aimed to compare the incremental time and cost burden of adding docetaxel to ARPI-based treatment among patients with mCSPC receiving chemotherapy-containing regimens (CCR) and non–chemotherapy-containing regimens (NCR) in the United States.

**Methods:**

Clinical data from community urology practices linked with claims data (1/1/2016–12/31/2023) were used to select patients initiating a CCR or NCR. Outcomes, including time spent managing mCSPC (days with prostate cancer-related resource utilization or management care) and healthcare costs, were compared between balanced cohorts using weighted multivariable Poisson and linear regressions.

**Results:**

126 CCR and 837 NCR patients (mean age 64.7 years, 52.6% White, 14.4% Black) were followed for a mean of 6.3 (CCR) and 6.8 (NCR) months. The CCR cohort spent on average 4.1 days per-patient-per-month (PPPM) managing mCSPC vs 3.3 days PPPM in the NCR cohort (rate ratio: 1.18; 95% confidence interval [CI]; 1.03, 1.34). Mean all-cause total healthcare costs were $17,833 PPPM in the CCR cohort and $11,527 PPPM in the NCR cohort (weighted adjusted cost difference: $6,184; 95% CI: 3,515, 8,517).

**Conclusions:**

Patients initiating a CCR experienced greater time burden managing mCSPC and higher healthcare costs than those initiating an NCR. These findings support counseling expressing these differences in burden in mCSPC treatment decision-making conversations.

## 1 Introduction

Prostate cancer (PC) is the second leading cause of cancer-related death among men in the United States (US) (1). Metastatic castration-sensitive prostate cancer (mCSPC), also known as metastatic hormone-sensitive prostate cancer, is an advanced stage of PC that is sensitive to hormone therapies (2). Chemotherapy (i.e., docetaxel) combined with androgen deprivation therapy (ADT) was the standard frontline treatment prior to the approval of oral androgen receptor pathway inhibitors (ARPIs), which are now widely used in combination with ADT for the treatment of mCSPC (3–5). Recently, intensifying treatment with a triple combination of chemotherapy, an ARPI (i.e., abiraterone acetate or darolutamide), and ADT has been recommended in practice guidelines as a treatment option for mCSPC (6–9).

With growing mCSPC treatment options, healthcare providers should consider various attributes of treatments that may impact patients’ outcomes and well-being (6). During cancer treatment selection, survival benefits and side effects of therapies are typically communicated with patients; however, the consideration of patients’ time and cost burden remains underacknowledged by oncologists (10, 11). Time burden of cancer treatment, also known as “time toxicity”, can be conceptualized as the time spent in coordinating care and in healthcare facility visits for receiving cancer-directed therapy, emergency care for side effects, hospitalization, and testing; the time spent during these visits also includes travel and wait times, which are often underrecognized (10, 11). Patient time burden is particularly relevant in advanced cancer settings when differences in survival benefits can be modest (or absent) between treatments, as the modest survival gain could be offset by the time lost to managing care (11, 12).

Given that chemotherapy with docetaxel has been associated with additional, potentially costly, clinical side effects in both clinical trial and real-world settings in mCSPC (13, 14), docetaxel-containing regimens may result in increased healthcare resource utilization (HRU) and costs, as well as greater time burden to manage care, relative to regimens without docetaxel. To date, however, data on the time burden and healthcare costs associated with chemotherapy-containing regimens (CCR) relative to non–chemotherapy-containing regimens (NCR) for mCSPC have been limited. This study aimed to compare the incremental HRU burden of chemotherapy intensification by assessing the number of days needed to manage PC care and healthcare costs among patients with mCSPC receiving ARPI-based treatment in the US.

## 2 Patients and methods

### 2.1 Data source

Clinical data from Precision Point Specialty Analytics (hereafter PPS) linked with administrative claims data from the Komodo Research Database (hereafter KRD) were used (01/01/2016–12/31/2023). The PPS database contains electronic medical record data collected as part of routine clinical care from >90 US community-based urology practices. The KRD contains claims data sourced from various payers and healthcare organizations covering >320 million US patients (see **Supplementary Methods** for more details). As all data are de-identified and Health Insurance Portability and Accountability Act compliant, approval from an institutional review board was not required.

### 2.2 Study design

A retrospective longitudinal analysis among propensity score–weighted cohorts of ARPI-naïve patients with mCSPC initiated on a CCR or an NCR was conducted. Patients were assigned to two mutually exclusive treatment cohorts based on the presence or absence of docetaxel intensification in the ARPI-based treatment: the *CCR cohort* included patients treated with the triplet regimen of docetaxel + abiraterone acetate or darolutamide + ADT, and the *NCR cohort* included patients treated with the doublet regimen of abiraterone acetate + ADT (as of the time of study conduct, darolutamide + ADT was not approved for treatment of mCSPC without the concurrent use of docetaxel (15)).

For the CCR cohort, the index date was the earliest of docetaxel initiation or first claim for abiraterone acetate or darolutamide on/after US Food and Drug Administration mCSPC indication approval dates for abiraterone acetate (02/07/2018 [high-risk mCSPC]) (16) or darolutamide (08/05/2022) (17). For the NCR cohort, the index date was the first claim for abiraterone acetate on/after the approval date for the high-risk mCSPC indication by the US Food and Drug Administration. The baseline period was the 6 months preceding the index date. The observation period spanned from the index date until the earliest of 12 months following the index date, discontinuation of the index ARPI or initiation of a new ARPI, initiation of docetaxel (NCR cohort only), end of continuous closed claims insurance eligibility, or end of data availability (including death).

### 2.3 Patient selection criteria

Adult patients were included if they i) had ≥1 paid pharmacy claim (in KRD) or dispensation (in PPS) for abiraterone acetate or darolutamide (first paid pharmacy claim or dispensation was defined as the ARPI initiation date); ii) had ≥1 medical or paid pharmacy claim or procedure for ADT observed ± 180 days around the ARPI initiation date; iii) (CCR cohort only) had ≥1 medical or paid pharmacy claim or procedure for docetaxel observed 90 days prior to or 180 days after the ARPI initiation date; iv) had mCSPC status on the index date, with <9 months between the date of metastasis and the index date (see **Supplementary Methods** for assessment details); and v) had ≥6 months of claims activity prior to (and including) the index date during a period of continuous closed claims insurance eligibility.

Patients were excluded if they had prior use of advanced PC-related medications (i.e., radiopharmaceutical therapy, chemotherapy [except docetaxel for the CCR cohort], apalutamide, enzalutamide, immunotherapy, or poly ADP-ribose polymerase inhibitors) any time prior to or on the index date; or if they became castration-resistant during the time between ARPI initiation and docetaxel initiation.

### 2.4 Study outcomes

Time spent managing mCSPC, defined as the number of days with PC-related HRU or PC management care, was assessed during the observation period. HRU categories included inpatient admission and days, emergency room (ER) visits, outpatient visits, and other services (e.g., dental or vision care, durable medical equipment services). PC management care included imaging, biopsy, chemotherapy management (i.e., iron/red blood cell transfusion, treatment with erythropoiesis-stimulating agents or granulocyte colony-stimulating factor [G-CSF]), prostate-specific antigen assessment, and genetic testing). When multiple HRU services for one patient on the same day were observed, these services only contributed one day. For inpatient admission, the number of days was calculated from the admission and discharge dates. In instances where the date of the claim differed from the date the service was received (e.g., laboratory tests or imaging), the date on which the service was received was used.

All-cause and PC-related HRU and healthcare costs were also assessed during the observation period. Total healthcare costs were the sum of medical costs (i.e., inpatient, ER, outpatient, and other services costs) and pharmacy costs. Separately, costs related to chemotherapy management (as defined above) in any setting were also assessed. Costs were inflated to 2023 US dollars using the medical care component of the Consumer Price Index.

All outcomes are reported per-patient-per-month (PPPM).

### 2.5 Statistical analyses

Overlap weighting, based on the propensity score, was used to account for differences in baseline characteristics between the CCR and NCR cohorts (**Supplementary Methods**) (18, 19). Balancing of baseline characteristics between both the non-weighted and weighted cohorts was assessed using standardized differences (<10% was considered well-balanced) (20).

The time spent managing mCSPC and HRU outcomes were compared between the weighted cohorts using weighted Poisson regression models with an offset to account for different follow-up times to calculate rate ratios (RRs). Cost outcomes were compared between the weighted cohorts using weighted ordinary least squares regression models. Non-parametric bootstrapping procedure with 500 replications was used to calculate 95% confidence intervals (CIs) and p-values for all models. All weighted regression models were further adjusted for baseline characteristics that remained imbalanced after weighting (**Supplementary Methods**).

## 3 Results

### 3.1 Baseline characteristics

**Figure 1:**
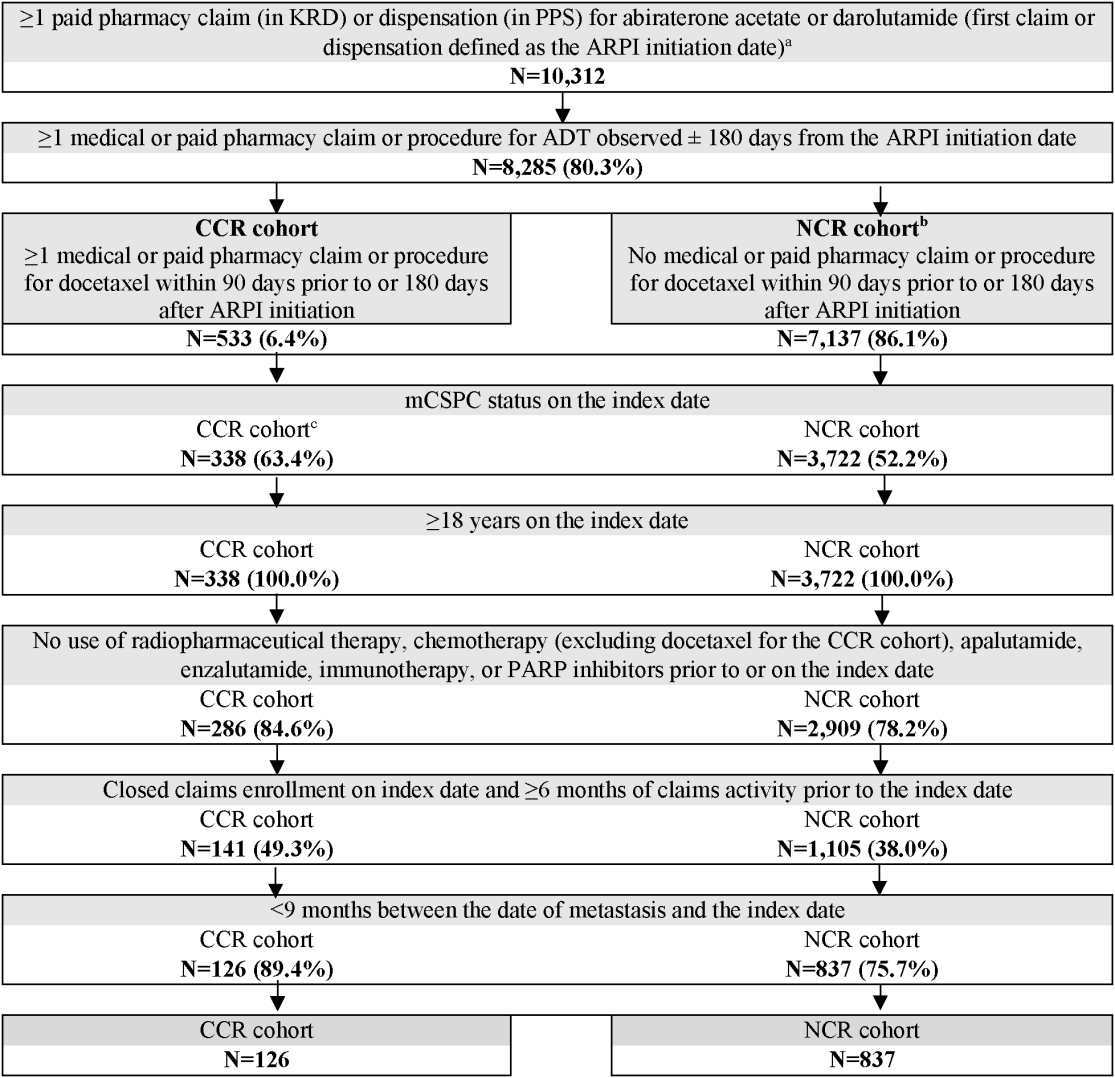
Patient selection flowchart. ADT, androgen deprivation therapy; ARPI, androgen receptor pathway inhibitor; CCR, chemotherapy-containing regimen; KRD, Komodo Research Database; mCSPC, metastatic castration-sensitive prostate cancer; NCR, non–chemotherapy-containing regimen; PARP, poly ADP-ribose polymerase; PPS, Precision Point Specialty; US, United States. a. ARPI initiation date on or after US Food and Drug Administration approval for mCSPC (i.e., 02/07/2018 for abiraterone acetate [high-risk mCSPC], 08/05/2022 for darolutamide). b. Only ARPI-naïve patients who initiated abiraterone acetate + ADT were included in the NCR cohort given as of the time of study conduct, darolutamide + ADT was not approved for treatment of mCSPC without the concurrent use of docetaxel. c. Patients were excluded if they became castration-resistant during the time between ARPI initiation and docetaxel initiation.

Overall, 126 patients were included in the CCR cohort and 837 in the NCR cohort (**Figure 1**). Baseline patient characteristics were generally well-balanced between the weighted cohorts, with standardized differences <10% (**Table 1**). In both the weighted CCR and NCR cohorts, mean age was 64.7 years, 52.6% were White patients, and 14.4% were Black or African American patients. Over 60% (61.9%) of patients were covered by commercial insurance, whereas 29.9% and 8.2% were Medicare and Medicaid beneficiaries, respectively. During baseline, most patients had bone metastasis (87.5% in both cohorts), about half had nodal metastasis (56.4% in CCR cohort; 42.7% in NCR cohort), and one-third had visceral metastasis (33.7% in both cohorts). Nearly 80% (78.5%) of patients in both cohorts had *de novo* metastasis and 54.6% had benign prostatic hyperplasia.

**Table 1:** Baseline characteristics.

Characteristics	Non-weighted population					Weighted population^a^				
	CCR (Docetaxel + Abiraterone Acetate/Darolutamide + ADT)		NCR (Abiraterone Acetate + ADT)			CCR (Docetaxel + Abiraterone Acetate/Darolutamide + ADT)		NCR (Abiraterone Acetate + ADT)		
	N=126		N=837		Standardized difference, %	N=126		N=837		Standardized difference, %
Age, mean ± SD [median]	64.1 ± 7.5 [63.3]		68.5 ± 9.7 [66.6]		50.2	64.7 ± 7.6 [64.5]		64.7 ± 9.3 [63.4]		0.0
Race, n (%)										
White	64	(50.8)	461	(55.1)	8.6	66	(52.6)	440	(52.6)	0.0
Black or African American	17	(13.5)	168	(20.1)	17.7	18	(14.4)	121	(14.4)	0.0
Other	16	(12.7)	128	(15.3)	7.5	17	(13.4)	112	(13.4)	0.0
Unknown	29	(23.0)	80	(9.6)	37.1	25	(19.6)	164	(19.6)	0.0
Geographic region, n (%)										
South	51	(40.5)	289	(34.5)	12.3	50	(40.1)	295	(35.2)	10.0
Midwest	36	(28.6)	259	(30.9)	5.2	36	(28.4)	258	(30.9)	5.4
Northeast	26	(20.6)	180	(21.5)	2.1	26	(21.0)	171	(20.4)	1.5
West	13	(10.3)	109	(13.0)	8.4	13	(10.5)	113	(13.5)	9.1
Payer, n (%)										
Commercial	81	(64.3)	379	(45.3)	38.9	78	(61.9)	518	(61.9)	0.0
Medicare	34	(27.0)	397	(47.4)	43.3	38	(29.9)	250	(29.9)	0.0
Medicaid	11	(8.7)	61	(7.3)	5.3	10	(8.2)	69	(8.2)	0.0
Metastasis type^b^, n (%)										
Bone	113	(89.7)	537	(64.2)	63.6	110	(87.5)	733	(87.5)	0.0
Nodal	71	(56.3)	432	(51.6)	9.5	71	(56.4)	357	(42.7)	27.7
Visceral	47	(37.3)	211	(25.2)	26.3	42	(33.7)	282	(33.7)	0.0
*De novo* metastasis^c^, n (%)	102	(81.0)	549	(65.6)	35.3	99	(78.5)	657	(78.5)	0.0
Benign prostatic hyperplasia, n (%)	71	(56.3)	418	(49.9)	12.9	69	(54.6)	457	(54.6)	0.0
PC-related total costs (PPPM)^d^, mean ± SD [median]	4,013 ± 5,000 [2,502]		2,809 ± 3,563 [1,530]		27.7	3,553 ± 4,138 [2,342]		3,553 ± 4,349 [1,860]		0.0

ADT, androgen deprivation therapy; CCR, chemotherapy-containing regimen; NCR, non–chemotherapy-containing regimen; PC, prostate cancer; PPPM, per-patient-per-month; SD, standard deviation.

a. Of note, the number of patients reported in this weighted population represents the sum of weights for the corresponding non-weighted patients, rounded to the nearest integer. The proportions displayed were calculated before the rounding and may be slightly different than if they were calculated based on rounded numbers.

b. Types of metastases were defined at any time prior to (and including) the index date. Types of metastases were not mutually exclusive.

c. *De novo* metastasis was defined as ≤180 days between first observed PC diagnosis and date of metastasis.

d. Costs were inflated to 2023 US dollars using the medical care component of the Consumer Price Index.

### 3.2 Time spent managing mCSPC and HRU

**Figure 2:**
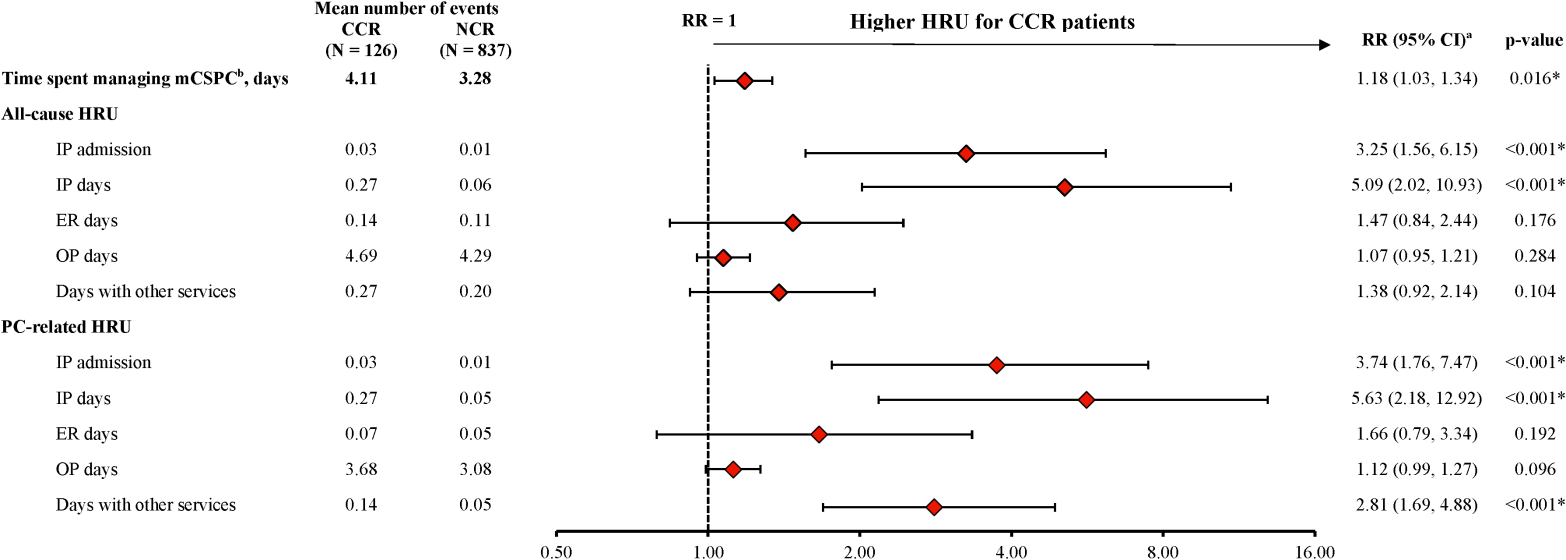
HRU among patients receiving CCR or NCR, PPPM. CCR, chemotherapy-containing regimen; CI, confidence interval; ER, emergency room; HRU, healthcare resource utilization; IP, inpatient; mCSPC, metastatic castration-sensitive prostate cancer; NCR, non–chemotherapy-containing regimen; OP, outpatient; PC, prostate cancer; PPPM, per-patient-per-month; RR, rate ratio. * p-value <0.05. a. The weighted model was adjusted for the following baseline variables: all-cause pharmacy costs, categorical age (≤70, 71-80, ≥81), time between metastasis and index date, baseline time spent managing mCSPC, erectile dysfunction, nodal metastasis and Quan-Charlson comorbidity index score. b. The time spent managing mCSPC is reported from the patients’ perspective, by days with a PC-related HRU or PC management care HRU outcome. When multiple HRU services for one patient on the same day were observed, these services only contributed one day. For inpatient stays, the number of days was calculated from the admission and discharge dates. In instances where the date of the claim differed from the date the service was received (e.g., laboratory tests or imaging), the date in which the service was received was used. All days were summed per person, and time spent managing mCSPC was reported PPPM.

Patients were observed for a mean of 6.3 months (median 6.0 months) in the CCR cohort and 6.8 months (median 6.7 months) in the NCR cohort. For the CCR cohort, a mean of 4.0 docetaxel infusions were observed per patient, with a mean of 22 days between successive infusions. During the observation period, the CCR cohort spent a mean of 4.1 days PPPM (median 3.6 days PPPM) managing mCSPC, compared with 3.3 days PPPM (median 2.4 days PPPM) in the NCR cohort (RR: 1.18, 95% CI: 1.03, 1.34; p=0.016; **Figure 2**).

The CCR cohort experienced a greater number of all-cause inpatient admissions (RR: 3.25, 95% CI: 1.56, 6.15; p<0.001) and inpatient days (RR: 5.09, 95% CI: 2.02, 10.93; p<0.001) compared with the NCR cohort during the observation period (**Figure 2**). Similar results were seen for PC-related inpatient admissions (RR: 3.74; 95% CI: 1.76, 7.47; p<0.001) and inpatient days (RR: 5.63; 95% CI: 2.18, 12.92; p<0.001). In addition, the number of PC-related days with other services was also greater in the CCR cohort than in the NCR cohort (RR: 2.81, 95% CI: 1.69, 4.88; p<0.001; **Figure 2**). The number of days spent on all-cause and PC-related ER visits trended higher in the CCR cohort relative to the NCR cohort, although the differences were not statistically significant (RR for all-cause ER days: 1.47, 95% CI: 0.84, 2.44; p=0.176; PC-related ER days: 1.66, 95% CI: 0.79, 3.34; p=0.192; **Figure 2**).

### 3.3 Economic burden

**Figure 3:**
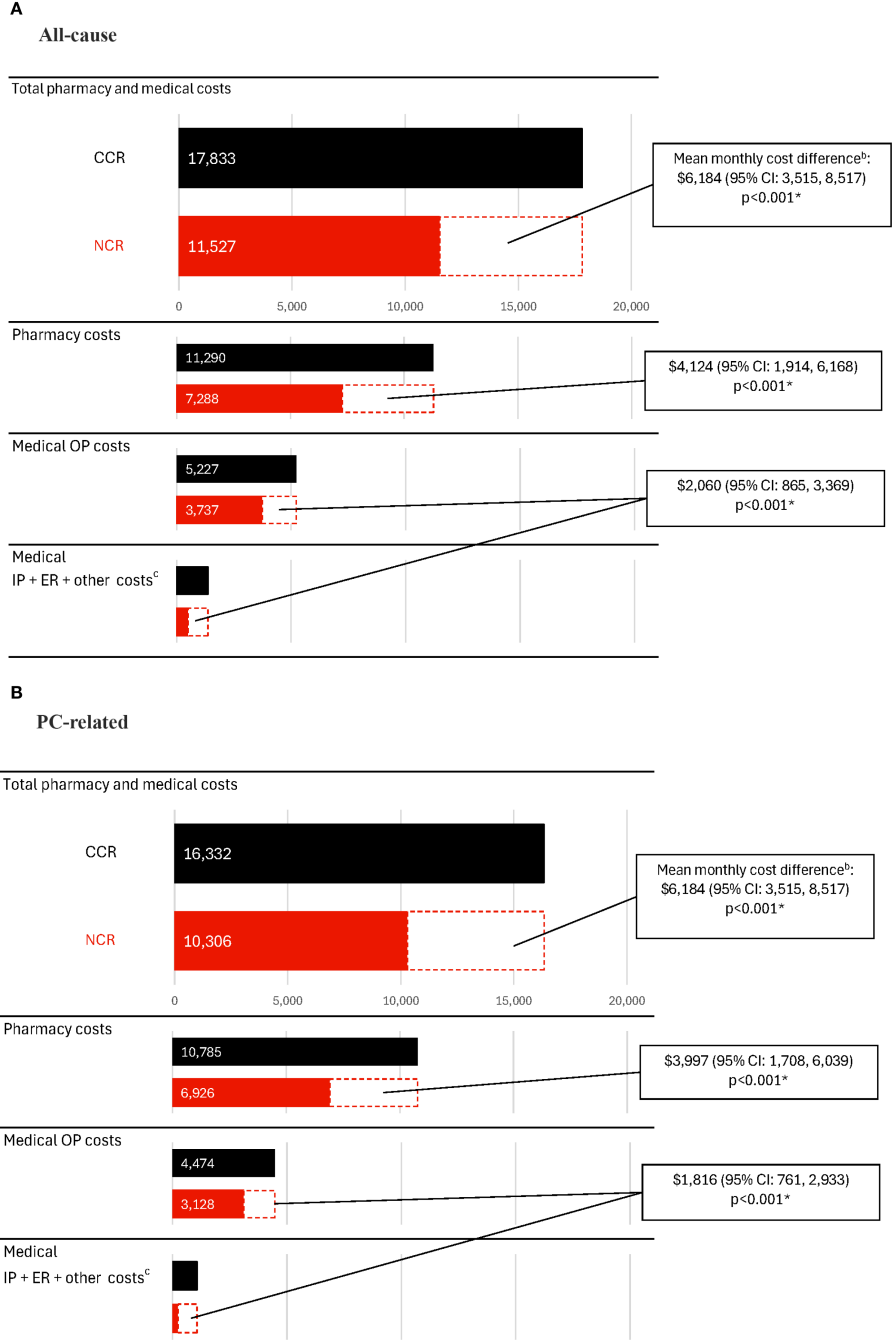
Healthcare costs among patients receiving CCR or NCR, PPPM^a^. **(A)** All-cause costs. **(B)** PC-related costs. CCR, chemotherapy-containing regimen; CI, confidence interval; ER, emergency room; IP, inpatient; mCSPC, metastatic castration-sensitive prostate cancer; NCR, non–chemotherapy-containing regimen; OP, outpatient; PC, prostate cancer; PPPM, per-patient-per-month; US, United States. * p-value <0.05. a. Costs were inflated to 2023 US dollars using the medical care component of the Consumer Price Index and were reported PPPM. b. The weighted model was adjusted for the following baseline variables: all-cause pharmacy costs, categorical age (≤70, 71-80, ≥81), time between metastasis and index date, baseline time spent managing mCSPC, erectile dysfunction, nodal metastasis and Quan-Charlson comorbidity index score. c. Other costs included durable medical equipment, dental, and vision care costs.

During the observation period, the CCR cohort incurred significantly higher healthcare costs compared with the NCR cohort (**Figure 3A**, **Supplementary Table 1**). The mean all-cause total healthcare costs were $17,833 PPPM in the CCR cohort and $11,527 PPPM in the NCR cohort (cost difference: $6,184; 95% CI: 3,515, 8,517; p<0.001). Medical costs constituted nearly 40% of the all-cause total costs and were $6,592 PPPM in the CCR cohort and $4,240 PPPM in the NCR cohort (cost difference: $2,060; 95% CI: 865, 3,369; p<0.001). All-cause pharmacy costs were $11,290 PPPM in the CCR cohort and $7,288 PPPM in the NCR cohort (cost difference: $4,124; 95% CI: 1,914, 6,168; p<0.001).

Similar trends in cost differences between the CCR and NCR cohorts were observed for PC-related costs (difference for PC-related total healthcare costs: $5,813, 95% CI: 3,135, 8,125; PC-related medical costs: $1,816, 95% CI: 761, 2,933; PC-related pharmacy costs: $3,997, 95% CI: 1,708, 6,039; all p<0.001; **Figure 3B**; **Supplementary Table 1**).

In the CCR cohort, chemotherapy management costs were $1,225 PPPM, driven largely by G-CSF use ($920 PPPM).

## 4 Discussion

In this retrospective analysis, during the first 12 months following ARPI-based treatment initiation, patients with chemotherapy intensification experienced 18% more days lost to mCSPC management, and incurred significantly greater healthcare costs, than those not treated with chemotherapy. For patients receiving a CCR, incremental costs of chemotherapy amounted to over $6,000 per month overall, and over $2,000 per month for medical costs, relative to those receiving an NCR.

To the best of our knowledge, this study is the first to quantify time burden associated with chemotherapy intensification in the context of ARPI-based treatment in mCSPC. Although time burden of cancer-related care, including chemotherapy, has been reported, few studies were specific to metastatic PC (21–23). One recent study in the metastatic castration-resistant PC setting found that chemotherapy was associated with more days spent with healthcare contact (e.g., for infusion, imaging, hospitalization, ER visits) compared with supportive care during the first quarter of the year prior to patients’ death, and such association was not observed with ARPIs (24). The results of that study highlight the time burden associated with chemotherapy for metastatic PC treatment, albeit in a different setting from the current study.

Time burden is most relevant for patients with advanced disease such as metastatic PC when life expectancy becomes limited (11). Indeed, research indicates that the consideration of both time and survival attributes could impact patients’ cancer treatment decisions (25). While clinical trials have demonstrated survival benefits of the triplet combination of docetaxel, ARPI (specifically abiraterone acetate and darolutamide), and ADT over docetaxel and ADT doublet regimens in patients with mCSPC (8, 9), a systematic review and meta-analysis of mCSPC trials found that the triplet combination of docetaxel, ARPI, and ADT, compared with the doublet combination without docetaxel, was not associated with improved overall survival (26). Real-world studies on the effectiveness of triplet therapy in mCSPC are emerging and have largely shown that the therapy is associated with high response rates and tolerable adverse event profiles (27–30), but survival data have been limited due to short and variable follow-up times and thus potential survival benefits over doublet regimens remain to be elucidated. Importantly, the current study provides insights on the additional time commitment required to manage chemotherapy and attend healthcare service visits among patients receiving a CCR relative to those receiving an NCR, highlighting the substantial incremental burden associated with chemotherapy intensification in mCSPC and the importance of informed discussions that include tradeoffs between increased time burden and potential survival benefit during treatment decision-making.

This study also found that patients with mCSPC incurred considerable incremental healthcare costs from chemotherapy intensification when receiving triplet versus doublet regimen. While the economic burden associated with mCSPC in relation to HRU and disease progression has been previously reported (31, 32), the cost findings in the current study shed light on the healthcare costs specifically attributable to the addition of docetaxel to ARPI-based treatment. In addition to greater pharmacy costs partially owing to the additional chemotherapy-related agents used in the triplet regimen such as G-CSF products, a sizable portion of the incremental total costs in patients receiving CCR was accounted for by additional medical costs, mainly the costs for outpatient visits and inpatient admissions. The significant increase in incremental healthcare costs observed in this study may be in part due to the management of side effects associated with chemotherapy (13, 14).

Notably, patients with chemotherapy intensification had more than three times the number of inpatient admissions and five times the number of inpatient days each month compared with those not treated with chemotherapy. These substantially increased rates in acute care may be in part due to unmanaged disease or treatment side effects requiring hospitalizations among patients receiving CCR (14). As studies have suggested that patients with metastatic PC generally prefer treatments that delay chemotherapy and require fewer additional hospital visits (33), the reduced inpatient admission rates and days associated with NCR therapies may help mitigate the stress and burden imposed on patients, as well as on the healthcare system (31).

Together, the substantial time and economic burden associated with chemotherapy intensification in ARPI-based treatment illustrated in this study underscore the importance of counseling expressing these differences in burden in decision-making conversations when selecting treatment for mCSPC. Strategies to incorporate the consideration of time burden in research and routine mCSPC care, such as the development and implementation of standardized metrics, are warranted to improve care quality (11, 34, 35).

The study findings should be considered with the following limitations. First, miscoding or misclassification in the clinical record or through the administrative claims may introduce selection and information biases despite efforts to balance the study populations. Second, overlap weighting and adjusted regression analyses could only adjust for measured covariates, and residual confounding may be present. Third, the cost component of the results may not be generalizable to healthcare systems outside of the US. Additionally, larger studies with more recent patient cohorts and longer follow-up are required to continue monitoring and evaluating the findings of this study for broader applicability.

## 5 Conclusions

In this real-world study, patients initiating an intensified ARPI-based treatment with docetaxel experienced greater time burden managing mCSPC and higher healthcare costs, including medical costs, than those initiating treatment without docetaxel. Treatment decision-making for mCSPC should include informed discussions considering time and cost burden of chemotherapy when included in treatment with an ARPI and ADT.

## Data Availability

The datasets presented in this article are not readily available because the data that support the findings of this study are from PPS Analytics and Komodo Health Solutions, and restrictions apply to the availability of these data, which were used under license for this study. Requests to access the datasets should be directed to https://portal.ppsanalytics.com/ or https://www.komodohealth.com/.
