# Supplementary material for: Time and cost burden associated with docetaxel in patients with metastatic castration-sensitive prostate cancer initiating an androgen receptor pathway inhibitor-based regimen
Source: Front Oncol. 2025 Sep 26;15:1650378. doi: 10.3389/fonc.2025.1650378 (PMC12512215; doi:10.3389/fonc.2025.1650378)
Supplement: Supplementary file 1 [file Supplementaryfile1.docx]

# Supplementary Materials

## Supplementary methods

### Data source

The Precision Point Specialty Analytics (hereafter PPS) contains electronic medical record data that include patient demographics and clinical variables such as laboratory test results, ARPI dispensation information, medication and procedure use (e.g., ADT, chemotherapies, imaging tests), and diagnoses. The Komodo Research Database (hereafter KRD) contains both open and closed insurance subsets and includes information on diagnoses and procedures received in inpatient and outpatient settings, along with prescription fills. In this study, only patients with closed portion medical and pharmacy claims were used to assess HRU and healthcare costs. Patient-specific tokens in the PPS and KRD datasets were created using machine learning-validated, de-identification technology to allow data from the PPS and KRD databases to be linked.

### Assessment of castration-sensitive status

The metastatic castration-sensitive prostate cancer (mCSPC) status was assessed using clinical indicators. The assessment of metastatic disease was defined based on bone, nodal, or visceral metastasis, identified through derived variables from the Precision Point Specialty Analytics (PPS) database or the International Classification of Diseases, 9^th^/10^th^ Revision, Clinical Modification (ICD-9/10-CM) diagnosis codes for metastasis identified in the PPS and Komodo Research Database. The earliest observed date from the PPS-derived variables or diagnosis codes for metastasis observed across both sources was used to define the initial metastasis date.

As there was no direct assessment for the castration sensitive status, patients without indicators for castration resistance were considered as castration sensitive. Castration resistance was assessed using a previously published algorithm incorporating presence of androgen deprivation therapy (ADT) and prostate-specific antigen (PSA) levels (1). Patients were considered to have castration-resistant prostate cancer (CRPC) if they met at least one of the following criteria:

- ≥1 indicator for CRPC based on provided variables in PPS
  - The indicator considered one of the following criteria: two consecutive rises in PSA while on continuous luteinizing hormone-releasing hormone therapy for >6 months, the presence of enzalutamide before it received a non-CRPC indication, manual data entry by a clinical user, the presence of the Z19.2 ICD-10-CM diagnosis code, and parsable encounter notes in the electronic medical records where a physician indicated that patients progressed to CRPC
- ≥1 diagnosis for castration resistant malignancy status
  - ≥1 claim with a diagnosis code ICD-10-CM: Z19.2 identified in PPS or Komodo
- Surgical castration and ≥1 rise in PSA from post-castration nadir
  - ≥1 record for bilateral orchiectomy observed at any time before the first observed ARPI used *and* ≥2 PSA test results at any time before the first observed ARPI used (including one nadir and one post-nadir) after the procedure, with ≥1 rise in PSA (of ≥25% with an absolute increase of ≥2 ng/mL) after nadir (i.e., the lowest PSA value observed between the procedures and the first ARPI claim)
- Medical castration and ≥1 rise in PSA from post-castration nadir
  - ≥90 days of continuous ADT use observed at any time before the first observed ARPI use *and* ≥2 PSA test results at any time before the first observed ARPI used (including one nadir and one post-nadir) within the same episode of continuous ADT, with ≥1 rise in PSA (of ≥25% with an absolute increase of ≥2 ng/mL) after nadir

### Propensity score weighting and regression model adjustments

The propensity score was obtained from a logistic regression model in which the index treatment cohort (i.e., CCR or NCR) was the dependent variable and the following baseline characteristics were independent variables: age, race, insurance type, bone metastasis, visceral metastasis, *de novo* metastasis (i.e., ≤180 days between first observed PC diagnosis and date of metastasis), benign prostatic hyperplasia and PC-related medical and pharmacy costs.

As some baseline characteristics remained imbalanced after weighting, all weighted regression models (i.e., Poisson regression and ordinary least squares regression models) were further adjusted for the following baseline characteristics: all-cause pharmacy costs, categorical age (≤70, 71-80, ≥81), time between metastasis and index date, baseline time spent managing mCSPC, erectile dysfunction, nodal metastasis and Quan-Charlson comorbidity index score.

## Supplementary Table 1. Follow-up healthcare costs^a^

| Mean ± SD [Median] | CCR  (N = 126) | NCR  (N = 837) | Weighted mean cost difference  (95% CI), p-value^b^ |
| --- | --- | --- | --- |
| **All-cause costs (PPPM)** |  |  |  |
| Total | 17,883 ± 11,382 [15,306] | 11,527 ± 13,070 [8,404] | 6,184 (3,515; 8,517), <0.001* |
| Pharmacy | 11,290 ± 10,120 [8,542] | 7,288 ± 11,324 [4,504] | 4,124 (1,914; 6,168), <0.001* |
| Medical | 6,592 ± 6,903 [4,870] | 4,240 ± 5,686 [2,590] | 2,060 (865; 3,369), <0.001* |
| Outpatient | 5,227 ± 4,156 [4,412] | 3,737 ± 5,321 [2,303] | 1,140 (301; 2,025), 0.004* |
| Inpatient | 979 ± 3,981 [0] | 302 ± 1,890 [0] | 702 (113; 1,367), 0.004* |
| Emergency room | 311 ± 1,372 [0] | 158 ± 615 [0] | 184 (-26; 473), 0.112 |
| Other^c^ | 75 ± 163 [0] | 43 ± 124 [0] | 34 (8; 63), 0.012* |
|  |  |  |  |
| **PC-related costs (PPPM)** |  |  |  |
| Total | 16,332 ± 11,174 [12,540] | 10,306 ± 12,772 [7,265] | 5,813 (3,135; 8,125), <0.001* |
| Pharmacy | 10,785 ± 10,141 [7,666] | 6,926 ± 11,271 [4,141] | 3,997 (1,708; 6,039), <0.001* |
| Medical | 5,547 ± 6,063 [3,858] | 3,380 ± 5,305 [1,952] | 1,816 (761; 2,933), <0.001* |
| Outpatient | 4,474 ± 3,674 [3,603] | 3,128 ± 5,067 [1,759] | 979 (168; 1,729), 0.016* |
| Inpatient | 844 ± 3,557 [0] | 201 ± 1,630 [0] | 656 (153; 1,281), <0.001* |
| Emergency room | 195 ± 1,184 [0] | 39 ± 192 [0] | 163 (12; 424), 0.020* |
| Other^c^ | 34 ± 91 [0] | 13 ± 57 [0] | 19 (3; 37), 0.016* |

CCR: chemotherapy-containing regimen; CI: confidence interval; mCSPC: metastatic castration-sensitive prostate cancer; NCR: non–chemotherapy-containing regimen; PPPM: per-patient-per-month; SD: standard deviation.

** p-value <0.05*

**Notes:**

a. Costs were reported in 2023 US dollars.

b. The weighted model was adjusted for the following baseline variables: all-cause pharmacy costs, categorical age (≤70, 71-80, ≥81), time between metastasis and index date, baseline time spent managing mCSPC, erectile dysfunction, nodal metastasis and Quan-Charlson comorbidity index score.

c. Other costs included durable medical equipment, dental, and vision care costs.

## Supplementary materials reference:

1. Freedland SJ, Ke X, Lafeuille MH, Romdhani H, Kinkead F, Lefebvre P, et al. Identification of patients with metastatic castration-sensitive or metastatic castration-resistant prostate cancer using administrative health claims and laboratory data. Curr Med Res Opin. 2021;37(4):609-22.
